# *WEScover*: selection between clinical whole exome sequencing and gene panel testing

**DOI:** 10.1186/s12859-021-04178-5

**Published:** 2021-05-20

**Authors:** In-Hee Lee, Yufei Lin, William Jefferson Alvarez, Carles Hernandez-Ferrer, Kenneth D. Mandl, Sek Won Kong

**Affiliations:** 1 Computational Health Informatics Program, Boston Childrens Hospital, 401 Park Drive, Mail Stop BCH3187, LM5528.4, Boston, MA 02115 USA; 2 Department of Pediatrics, Harvard Medical School, Boston, MA 02115 USA; 3 Department of Biomedical Informatics, Harvard Medical School, Boston, MA 02115 USA; 4 Agios Pharmaceuticals, Boston, MA USA; 5 Centre Nacional dAnlisi Genmica (CNAG-CRG), Barcelona, Spain

**Keywords:** Genetic testing, False negative, Coverage, Whole exome sequencing, Gene panel testing

## Abstract

**Background:**

Whole exome sequencing (WES) is widely adopted in clinical and research settings; however, one of the practical concerns is the potential false negatives due to incomplete breadth and depth of coverage for several exons in clinically implicated genes. In some cases, a targeted gene panel testing may be a dependable option to ascertain true negatives for genomic variants in known disease-associated genes. We developed a web-based tool to quickly gauge whether all genes of interest would be reliably covered by WES or whether targeted gene panel testing should be considered instead to minimize false negatives in candidate genes.

**Results:**

*WEScover* is a novel web application that provides an intuitive user interface for discovering breadth and depth of coverage across population-scale WES datasets, searching either by phenotype, by targeted gene panel(s) or by gene(s). Moreover, the application shows metrics from the Genome Aggregation Database to provide gene-centric view on breadth of coverage.

**Conclusions:**

*WEScover* allows users to efficiently query genes and phenotypes for the coverage of associated exons by WES and recommends use of panel tests for the genes with potential incomplete coverage by WES.

**Supplementary Information:**

The online version contains supplementary material available at 10.1186/s12859-021-04178-5.

## Background

As the cost of whole exome sequencing (WES) drops, WES is replacing targeted gene panel testing [1, 2]. WES, for example, is superior in measuring the ever-growing number of driver and passenger mutations in diverse genes across different cancer types as well as increasing awareness of oligogenic contribution to most genetic disorders [3]. However, WES is not capturing all exons in clinically implicated genes in the human genome [4, 5] and whole genome sequencing (WGS) faces a similar challenge for some genes including highly polymorphic ones. As such, population-scale aggregation of WES and WGS clearly shows limited breadth of coverage for some clinically implicated genes [4, 6].

Wang and colleagues found that a hereditary eye disease enrichment panel could identify pathogenic and likely pathogenic mutations in 41.2% of patients with inherited retinal dystrophies compared to 33.0% by WES [7]. In some cases, WES did not capture pathogenic variants in patients with inherited retinal diseases and candidate gene panels could suggest genetic causes [8]. Another study showed that a target-enriched exome sequencing approach was able to detect 99.7% known genetic variants responsible for neuromuscular disorders, comparing to 97.1% and 99.2% identified by two different WES analyses [9]. Interestingly, a cost analysis of next-generation sequencing using Illumina platforms showed that estimated costs per sample for targeted gene panels (333) were less than half of WES (792) [10]. Therefore, gene panel testing, whether for a single gene or for hundreds of candidate genes, is still a clinically useful measure when false negatives due to suboptimal coverage of WES and WGS are likely.

Yet it is difficult to predict whether the exons that are known to harbor disease-associated variants would be covered with sufficient per-site depth of coverage to reliably call variants or not.There have been efforts to identify regions or genes poorly covered by targeted panels or WES. ExomeSlicer provides per-exon depth of coverage based on 1,932 clinical exome sequencing samples so that users can identify regions with incomplete coverage for genes of interest [11]. Ebbert and colleagues systemically investigated the genesincluding disease genesthat were difficult to analyze with standard short-read sequencing technologies [12]. These tools provide useful measures on which genes might not be sufficiently covered by WES but lack means to suggest alternatives.

*WEScover* provides the advantage of summarizing coverage information on clinically implicated genes along with the information of gene panel tests for the genes. It can provide a basis to recommend the use of gene panel tests for the genes that are poorly covered by WES. Also, *WEScover* provides WES coverage stratified by continental-level population, highlighting population-specific differences in exome coverage. With a self-reported ancestry of the patient, users could find the coverage of a given gene among the matching population group, compared to other datasets such as Genome Aggregation Database (gnomAD) project [13] that only provides global mean coverage across all exomes. Links to gnomAD are also provided such that global coverage levels across large scale of samples can be checked.

### Implementation

*WEScover* is developed to assist decision making for biomedical investigators by providing empirical measure of breadth and depth of coverage in WES for genes of interest. Users can find global coverage summary of the exomes from the 1000 Genomes Project (1KGP) phase 3 data [14] (N=2,504) as well as between-population differences. For each gene, *WEScover* also provides a list of related genetic tests from the National Institutes of Health Genetic Testing Registry (GTR) [15] so that investigators can quickly search for alternatives when the gene may not be well-covered by WES.

#### Coverage metric in WEScover

The average read depth, the most widely used coverage metric, describes how many times each locus is supported by effectively aligned short-reads in WES on average. However, given the variance in the efficiency of exon capture baits, some coding regions are incompletely covered even though the average read depth is sufficiently high for the majority of exons [4]. Then, the absence of genetic variants could include false negatives. To address this issue, *WEScover* provides breadth of coverage at different levels of depth of coverage for each gene.

**Fig. 1:**
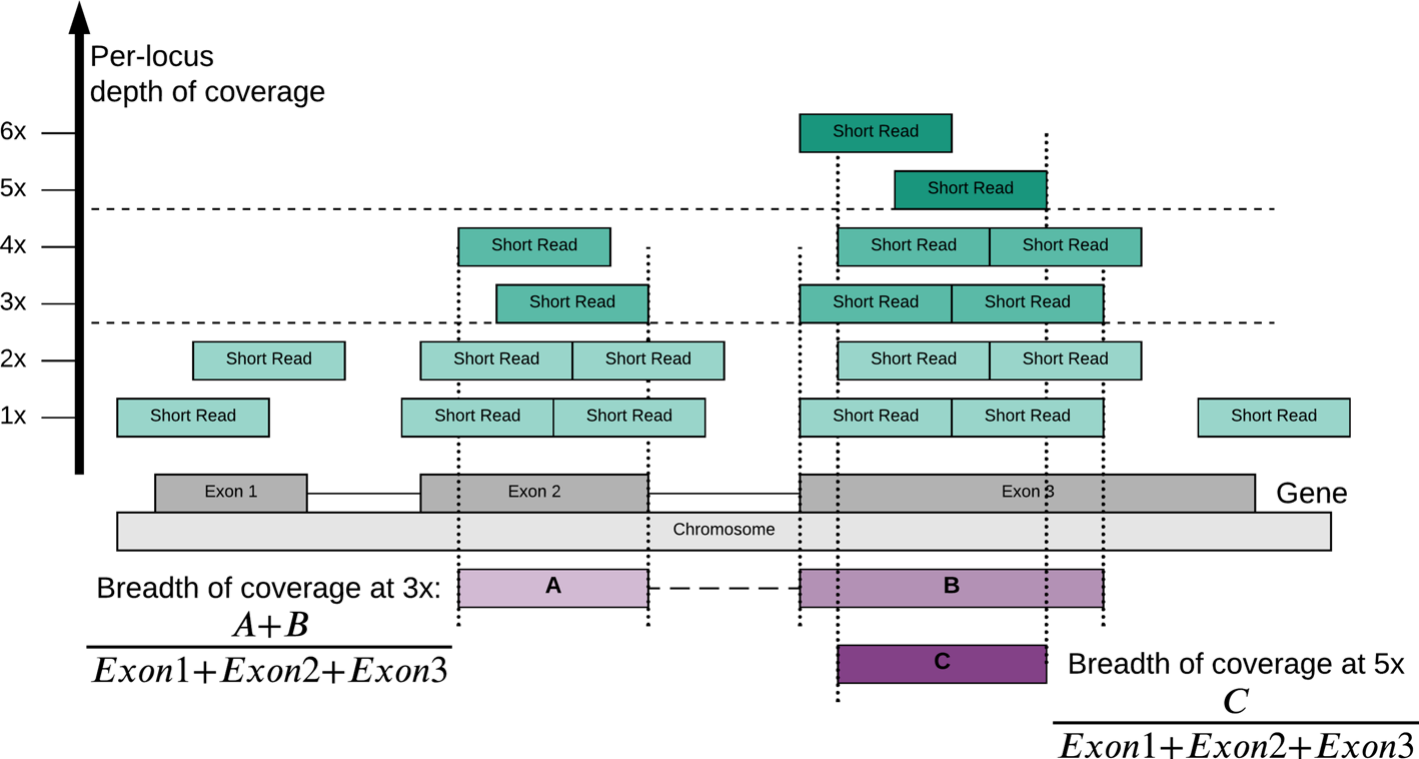
An illustration of breadth of coverage for a gene. It shows an example of a gene consisting of three exons (Exon 1, Exon 2, Exon 3). The short reads mapped to each exon are shown above the corresponding exons. Per-locus depth of coverage is equivalent to the count of short reads above each position in exon. Boxes A and B denote regions (positions) in the gene, where every locus has 3 or more reads. The breadth of coverage at 3is calculated as proportions of A and B within the gene (shown in the lower-left). Likewise, the breadth of coverage at 5is calculated as proportion of box Cevery locus has 5 or more readsin gene (shown in lower-right)

The breadth of coverage for a gene model is calculated as a proportion of protein coding sequences where the read depth is above a given threshold compared to total length of exons. For a gene with protein coding sequences of 300 base pairs (bps), the breadth of coverage at 10for the gene is 90% if the read depths for 270 out of 300 bps are above 10x. The breadth of coverage varies by the target level of read depth at each position and decreases as a higher depth of coverage is required. Figure1 illustrates the breadth of coverage at different read depth levels. *WEScover* calculates the breadth of coverage for each of different transcript models for a protein coding gene. The list and coordinates for all genes and transcripts are based on the Consensus Coding Sequence (CCDS) [16] (we used release 15 and 21 for human reference genome assembly version 37 (GRCh37) and 38 (GRCh38), respectively).

#### Global coverage and variation across populations

We calculated breadth of coverage for each gene at 8 different levels for read depths 5x, 10x, 15x, 20x, 25x, 30x, 50and 100x using the exomes from the 1000 Genomes Project (1KGP) phase 3 [14]. We used two sets of alignment files mapped to two human reference genome assemblies: GRCh37 and GRCh38. *WEScover* shows the average breadth of coverage across exomes in the 1KGP, as well as minimum and maximum values in 1KGP. *WEScover* also provides average breadth of coverage for each of the 5 population groups in 1KGP: Africa (AFR), American (AMR), East Asian (EAS), European (EUR), and South Asian (SAS). Each population may have different sequence context across the genome which affects exome capture efficiency and is reflected, in turn, in breadth and depth of coverage. The statistics from one-way ANOVA test, KolmogorovSmirnov test and Tukeys Honest Significant Difference test were provided to compare the average breadth of coverage among populations.

**Fig. 2:**
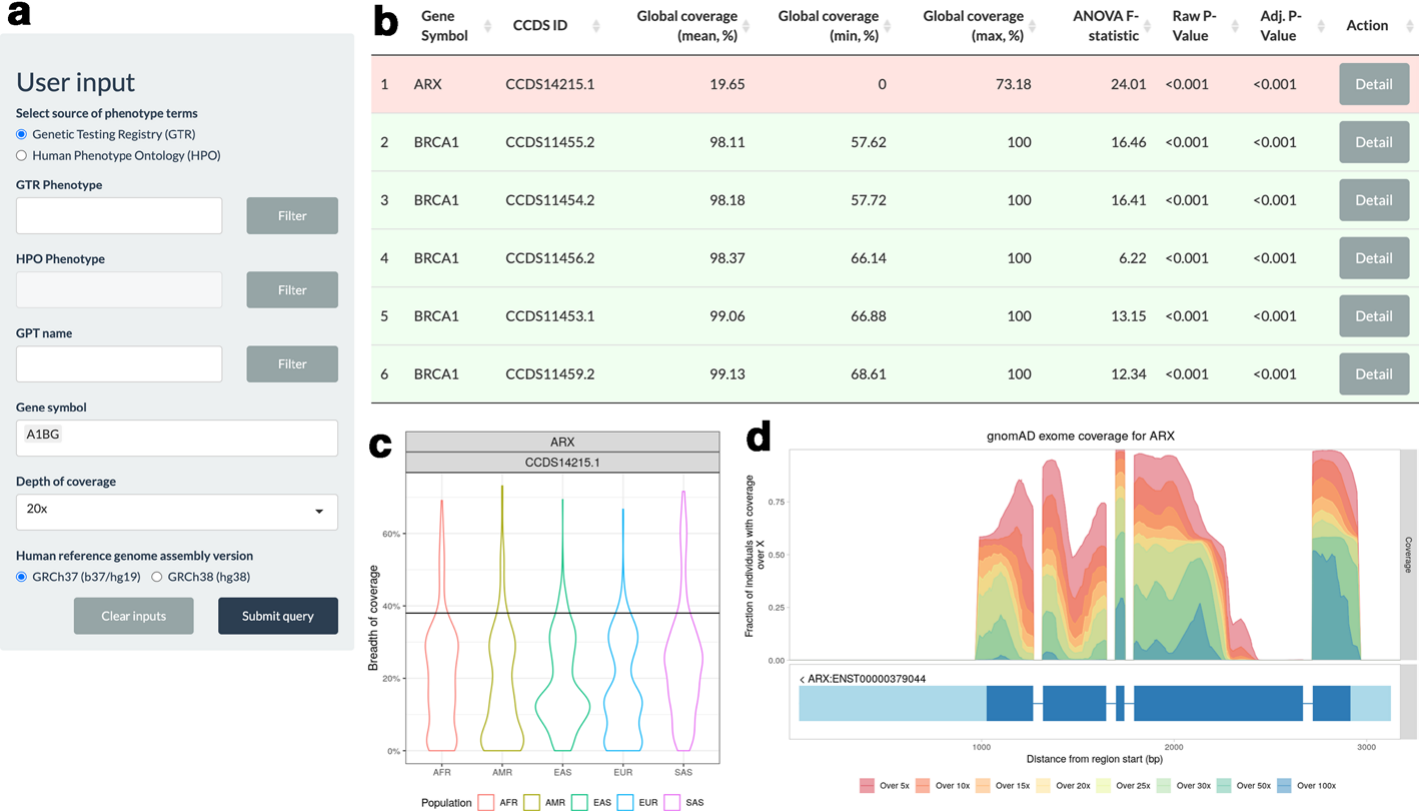
Query interface and outputs in *WEScover*. **a** The initial screen for user query. Users can specify genes of interest (Gene symbol) directly. Otherwise, phenotype (GTR Phenotype or HPO Phenotype) or gene panel test name (GPT name) can be used to search for related genes. Lastly, the expected depth of coverage level (Depth of coverage) and human genome reference assembly version used in calculating coverage (Human reference genome assembly version) need to be selected. **b** The result summary screen for the selected genes. Clicking Detail button (under the column Action) opens a window with more information such as breadth of coverage per continent-level population, its distribution in different populations (**c**), coverage value from gnomAD exomes (**d**), differences across populations, and list of gene panels including the transcript for selected gene. **c** The violin plot shows the distribution of coverage metrics from 1KGP exomes in each of the five continent-level populations. The black horizontal line denotes the global average value from gnomAD exomes. **d** The coverage plot (upper part) shows the per-locus coverage metric from gnomAD exomes across the gene. The per-locus coverage metric values are shown at various levels from 5x (red) to 100x (blue). The transcript(s) for the gene is shown beneath the coverage plot: light blue blocks for untranslated regions and dark blue blocks for exons. To highlight the coverage on exons, the introns are scaled down to the same length. The lengths of exons are maintained. It may show multiple transcripts for the gene, even if a single CCDS ID was selected

In an effort to have coverage data out of a larger collection of exomes and diverse exome capture kits, we made use of the coverage across 125,748 exomes available from gnomAD release 2.1. However, we were not able to calculate breadth of coverage from gnomAD exomes because of the lack of individual-level coverage data. Instead gnomAD provided the coverage summary, the proportion of samples over the given read depth at each locus, which we utilized to visualize the depth and extent of coverage of the gene (Fig.2d).

#### Gene panel testing as an alternative to WES

We collected the registered genetic tests listed in the National Institutes of Health Genetic Testing Registry (GTR) [15] to inform users of available genetic tests. Additionally, *WEScover* enables users to query phenotype to list candidate genes by integrating associated Human Phenotype Ontology (HPO) terms [17] for each genetic test from GTR. As of writing, a total of 59,928 genetic tests for both clinical and research usage in GTR (last accessed on Feb. 28^th^, 2021) were compiled in *WEScover*, including 32,390 CLIA-certified ones. A total of 6,097 putative disease-associated genes were linked to one or more of registered tests.

## Results

Using the relationship between phenotypes listed either in GTR or HPO, genetic test names from GTR and genes, we created a database and a query interface using R Shiny package [18]. The initial query interface allows users to enter phenotype, genetic test name (retrieved from the GTR website), or official gene symbol(s) of interest (Fig.2a). The phenotype can either be as listed in GTR or be standard terms from HPO. It also provides the choice of target depth of coverage: 5x, 10x, 15x, 20x, 25x, 30x, 50x, and 100x. As default choice, we use breadth of coverage at>20x a threshold sufficient to achieve 99% sensitivity for detecting single nucleotide variants [19]. Finally, users can also choose the human reference genome assembly version: GRCh37 and GRCh38 (latest). For each gene matching the query, the global mean of breadth of coverage along with its maximum and minimum values are shown in a table in an ascending order of global means (Fig.2b). We also perform a one-way analysis of variance to test differences between coverage means of populations and report the test statistics and p-values in this table. The button at the end of each row opens a window containing further details about the coverage of the gene. The panel first shows a table with the mean breadth of coverage stratified by continent-level populations. The second tab shows a violin plot for the breadth of coverage stratified by continent-level populations (Fig.2c). We also provided the mean gnomAD coverage metric (i.e., mean fraction of samples over X read depth across every position of the gene) for comparison with 1KGP exomes. Although the mean gnomAD coverage metric measures different value based on larger scale of samples across diverse exome platforms, it correlates well with the mean breadth of coverage (see Additional file 1). A plot for coverage at each genomic position of the selected gene, based on gnomAD coverage data, is shown next to the violin plot (Fig.2d). Additionally, we provide two results from tests of differences between each pair of populations: KolmogorovSmirnov test to compare between cumulative distributions, and Tukeys Honest Significant Difference test for pairwise comparison of means. Lastly, the panel reports all genetic tests involving the gene. Insufficient coverage in both projects, 1KGP and gnomAD, should inform the user that the candidate genes may not be sufficiently covered in WES and that targeted gene panel tests should be considered to minimize potential false negatives.

**Fig. 3:**
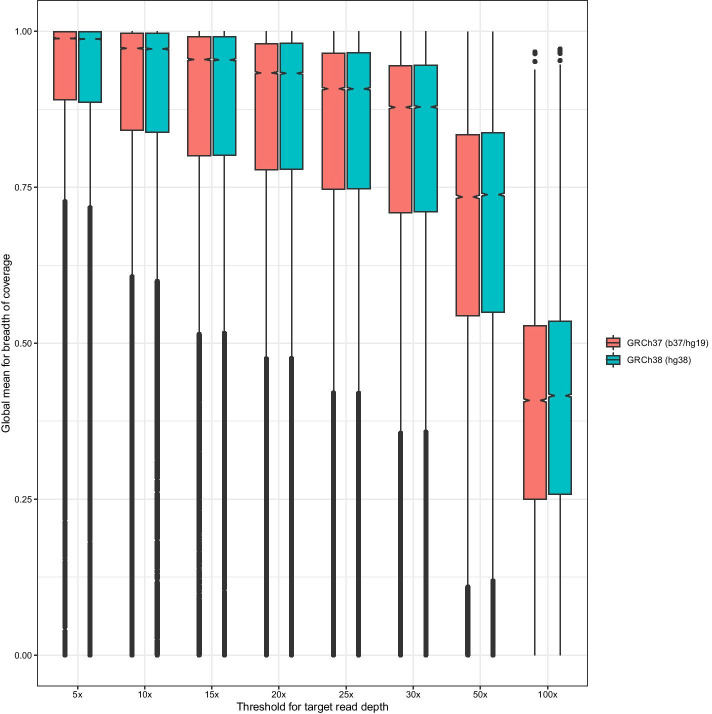
Distribution of mean breadth of coverage across various target read depths and genome assembly versions. The y-axis represents the average breadth of coverage across 1KGP exomes, for each gene. Each boxplot represents the distribution across all CCDS genes at read depths from 5to 100x. At each read depth, the distributions of values from each genome assembly versions (GRCh37 and GRCh38) are shown as separate boxplots. The overall breadth of coverage levels of genes is highest at 5and decreases as read depths are increased. Given the same read depth level, little difference is observed between genome assembly versions. Lower tails of boxplotsespecially at low read depths as 5or 10xindicate genes with poor breadth of coverage in most of 1KGP exomes

We further investigated the distribution of breadths of coverage at each per-locus target depth and human reference genome assembly versions (Fig.3). The median across all genes for global mean breadth of coverage at 20was 93.3%; that is, for majority of CCDS genes, 93.3% of gene was covered by 20 or more reads on average exomes. Due, in part, to the older design of exome capture targets in the 1KGP exomes, the breadth of coverage values in *WEScover* are better be taken as lower bounds. The trends of distribution were consistent across genome assembly versions in spite of the differences between CCDS releases. Of note, genes with very low (<10%) mean breadth of coverage were observed across all cases, even at low depths such as 5or 10x, suggesting that the exome capture targets for 1KGP did not cover all genes and their exons in the CCDS releases that we used in *WEScover*. These genes can be easily identified by checking coverage metric values from gnomAD exomes. If a gene is sufficiently covered by more recent exome data, it would have good coverage value among gnomAD exomes. Thus, *WEScover* shows both the mean gnomAD coverage metric and coverage plot over exons of the gene. We encourage users to check gnomAD browser for the genes with suboptimal coverage in *WEScover* before committing to gene panel testing.

There are two limitations of utilizing *WEScover*. Firstly, the breadth of coverage value (as well as gnomAD coverage metric) is not normalized for the factors generally contributing to exome coverage such as sequence context and GC contents. Such factors vary widely between genes and comparison of the values for one gene with another is beyond the proposed use of *WEScover*. Secondly, *WEScover* focuses on gene-level breadths of coverage and does not provide ways to search for specific variants and regions within genes.

## Conclusions

WES and WGS provide comprehensive evaluation of diverse types of genomic variants in various conditions. However, users must be informed regarding possible false negatives due to incomplete breadth and depth of coverage, ideally from sequencing vendors. In such cases, a targeted gene panel test should be considered as a primary choice over the others. *WEScover* can guide users as to whether WES is appropriate for testing the genes of interest. Considering that many laboratories, especially clinical testing facilities, are slow in transition from the previous genome build (GRCh37), *WEScover* supports coverage summary for both GRCh37 and GRCh38. Together with information from GTR, which provides transparent and comprehensive list of genetic tests with indications, users can make an informed decision for testing genes prior to ordering genetic tests in clinical settings.

## Availability and requirements

Project name: *WEScover.*

Project home page: https://tom.tch.harvard.edu/shinyapps/WEScover/

Project source code: https://github.com/bch-gnome/WEScover

Operating system: Platform independent.

Programming language: R Shiny.

Other requirements: *WEScover* requires the following R packages: *shiny*, *shinythemes*, *DT*, *ggplot2*, *shinyjs*, *shinyBS*, *reshape2*, *RColorBrewer*, *fst*, *data.table*, *wiggleplotr*, *patchwork*, *ggpubr*, *dplyr* and *corrplot*.

License: MIT.

Any restrictions to use by non-academics: None.

## Supplementary Information

**Additional file 1** Portable Network Graphics. Comparison between exome coverage metrics for 1000 Genomes Project (1KGP) and for gnomAD. Each panel shows coverage metrics for genes (based on CCDS release 15) measured with the chosen read depth (X): X=5x, 10x, 15x, 20x, 25x, 30x, 50x, and 100x. At each panel, x-axis represents the breadth of coverage for a gene (the fraction of gene which have X or higher read depth at a position) averaged over 2,504 exomes from 1KGP. On the other hand, y-axis shows the gnomAD exome coverage metric for a locus (the fraction of gnomAD exomes which have X or higher read depth at a position) averaged over all exons in a gene. Both values correlate well while the metric for gnomAD tends to have higher value than that for 1KGP. Also note that part of CCDS genes not included as exome target region for 1KGP have good metric value (>0.9) with gnomAD exomes (dots with x=0).

## Data Availability

Breadth of coverage data stratified by continent-level populations from 1000 Genomes Project (either in GRCh37 or GRCh38) are available for downloading on https://tom.tch.harvard.edu/shinyapps/WEScover/ under the Data tab.

## Abbreviations

WES: Whole exome sequencing
WGS: Whole genome sequencing
gnomAD: Genome aggregation database
GTR: Genetic testing registry
CCDS: Consensus coding sequence
1KGP: 1000 Genomes project
HPO: Human phenotype ontology
