# Supplementary figures and images for: WEScover: selection between clinical whole exome sequencing and gene panel testing
Source: BMC Bioinformatics. 2021 May 20;22:259. doi: 10.1186/s12859-021-04178-5 (PMC8139020; doi:10.1186/s12859-021-04178-5)

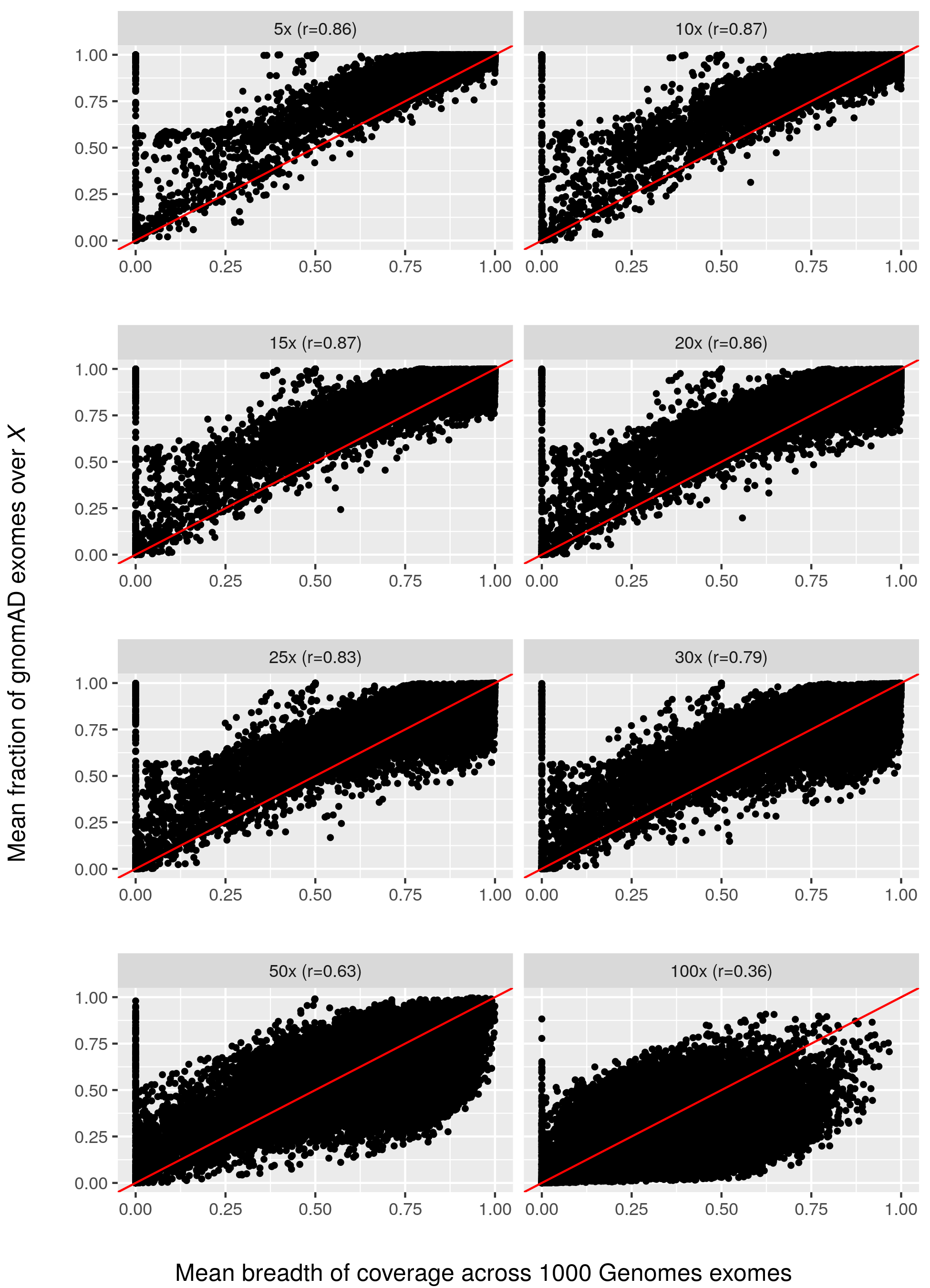

Supplement: Supplementary file 1 — Additional file 1 Portable Network Graphics. Comparison between exome coverage metrics for 1000 Genomes Project (1KGP) and for gnomAD. Each panel shows coverage metrics for genes (based on CCDS release 15) measured with the chosen read depth (X): X=5x, 10x, 15x, 20x, 25x, 30x, 50x, and 100x. At each panel, x-axis represents the breadth of coverage for a gene (the fraction of gene which have X or higher read depth at a position) averaged over 2,504 exomes from 1KGP. On the other hand, y-axis shows the gnomAD exome coverage metric for a locus (the fraction of gnomAD exomes which have X or higher read depth at a position) averaged over all exons in a gene. Both values correlate well while the metric for gnomAD tends to have higher value than that for 1KGP. Also note that part of CCDS genes not included as exome target region for 1KGP have good metric value (>0.9) with gnomAD exomes (dots with x=0). [file 12859_2021_4178_MOESM1_ESM.png]
